# Gadolinium enhanced magnetic resonance mean voxel signal intensity within the left ventricular myocardium changes prior to ejection fraction drop after receipt of cardiotoxic chemotherapy

**DOI:** 10.1186/1532-429X-11-S1-P80

**Published:** 2009-01-28

**Authors:** Jimmy C Lightfoot, Ralph B D'Agostino, Craig A Hamilton, William C Little, Frank M Torti, Nancy D Kock, Mike Robbins, W Gregory Hundley

**Affiliations:** Wake Forest University, Winston-Salem, NC USA

**Keywords:** Doxorubicin, Left Ventricular Ejection Fraction, Cardiac Magnetic Resonance, Cardiac Magnetic Resonance Image, Left Ventricular Myocardium

## Objective

To determine if serial measures of gadolinium (GD) signal intensity acquired with cardiac magnetic resonance (CMR) during receipt of cardiotoxic doxorubicin (DOX) chemotherapy are associated with left ventricular ejection fraction (LVEF).

## Methods

**Figure 1:**
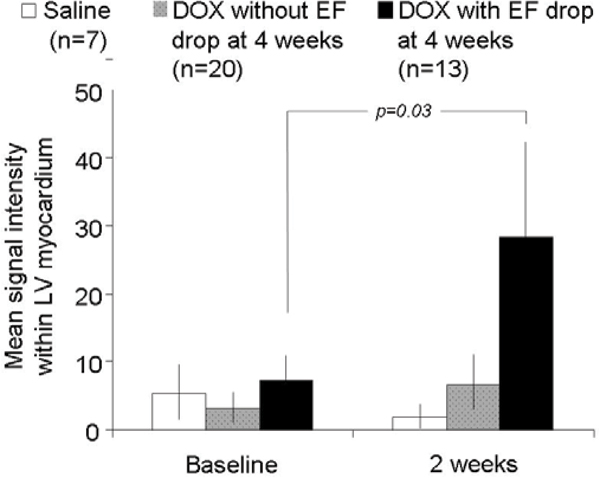
Figure 1

40 Sprague-Dawley rats were divided into 3 groups receiving weekly doses of: normal saline [NS] (n = 7), 1.5 mg/kg DOX (n = 19), or 2.5 mg/kg DOX (n = 14). 1.5 T CMR images of LVEF and myocardial GD signal were acquired before and at 2 and 4 weeks after DOX treatment. LVEF was determined from a multi-slice short axis cine acquisition; signal intensity was obtained from an inversion recovery (IR) mid-LV slice. Analysis of variance models were fit with group or LVEF drop status (>10% drop in LVEF from baseline yes/no, or NS) as factors. RESULTS: LVEF was similar among the 3 groups at baseline (p = 0.27), and at 2 weeks, (73+5%, NS; 74+5%,1.5 mg/kg DOX; 71+6%,2.5 mg/kg DOX). At 4 weeks, LVEF after NS was unchanged (77 ± 7%, p = 0.93), but there was a >10% drop in LVEF in 3/19 in the 1.5 mg/kg DOX, and 10/14 in the 2.5 mg/kg DOX groups: LVEF was 77 ± 7% in non-dropped and 64 ± 8% in dropped groups at 4 weeks (p < 0.001). LVEF remained ≥70% in all animals at 2 weeks. In animals dropping their LVEF at 4 weeks, the GD signal within the LV myocardium was elevated relative to baseline at week 2 (2 weeks prior to LVEF drop – Figure 1); in animals receiving NS or DOX without LVEF drop, GD was not elevated at 2 weeks.

## Conclusion

These data suggest gadolinium CMR may be useful to monitor for doxorubicin chemotherapy cardiotoxicity: low serial measures of GD intensity forecast no LVEF drop, whereas an increase in GD signal intensity forecasts a clinically important drop in LVEF.

